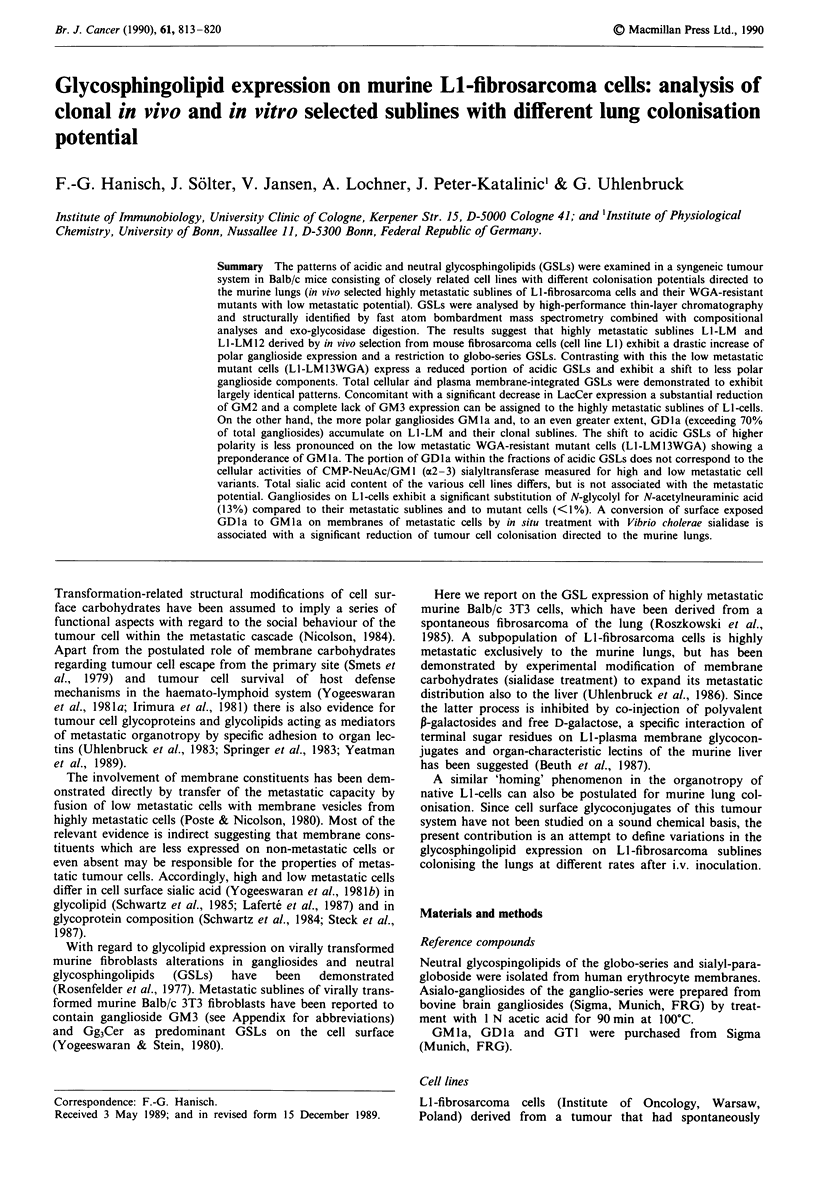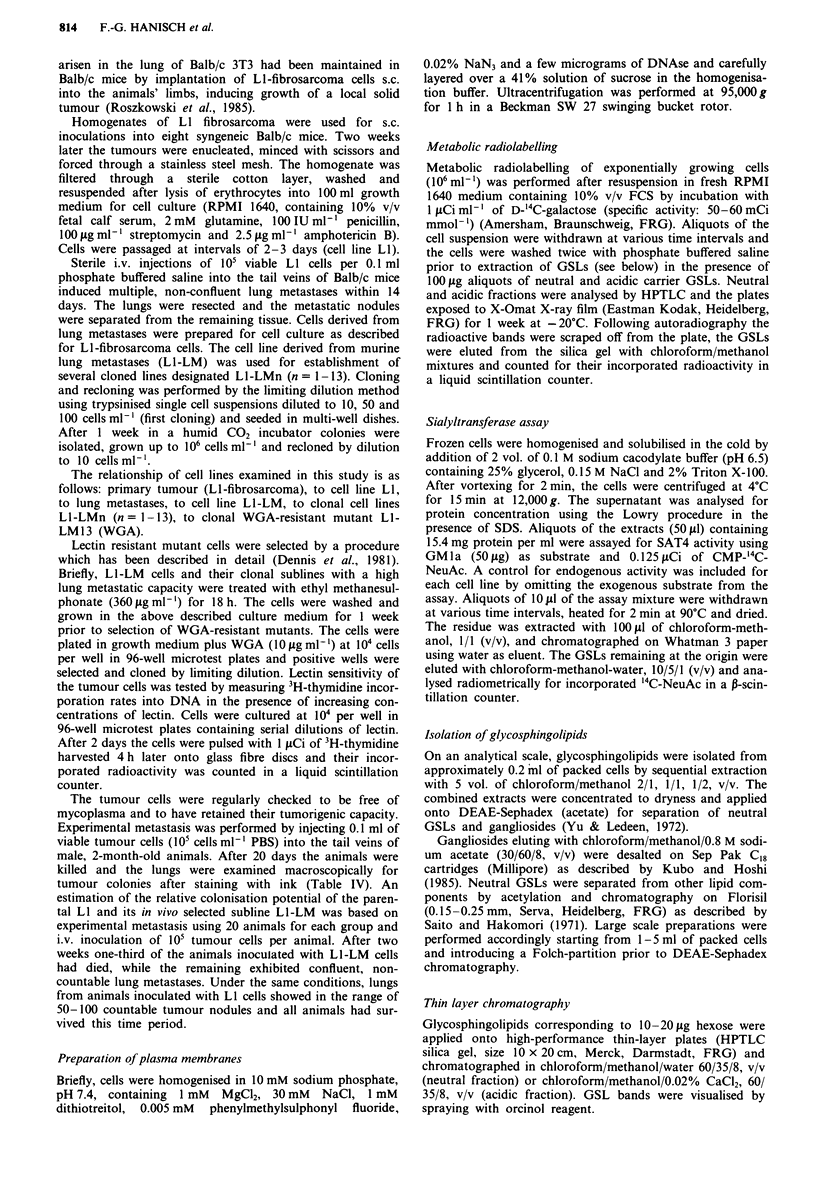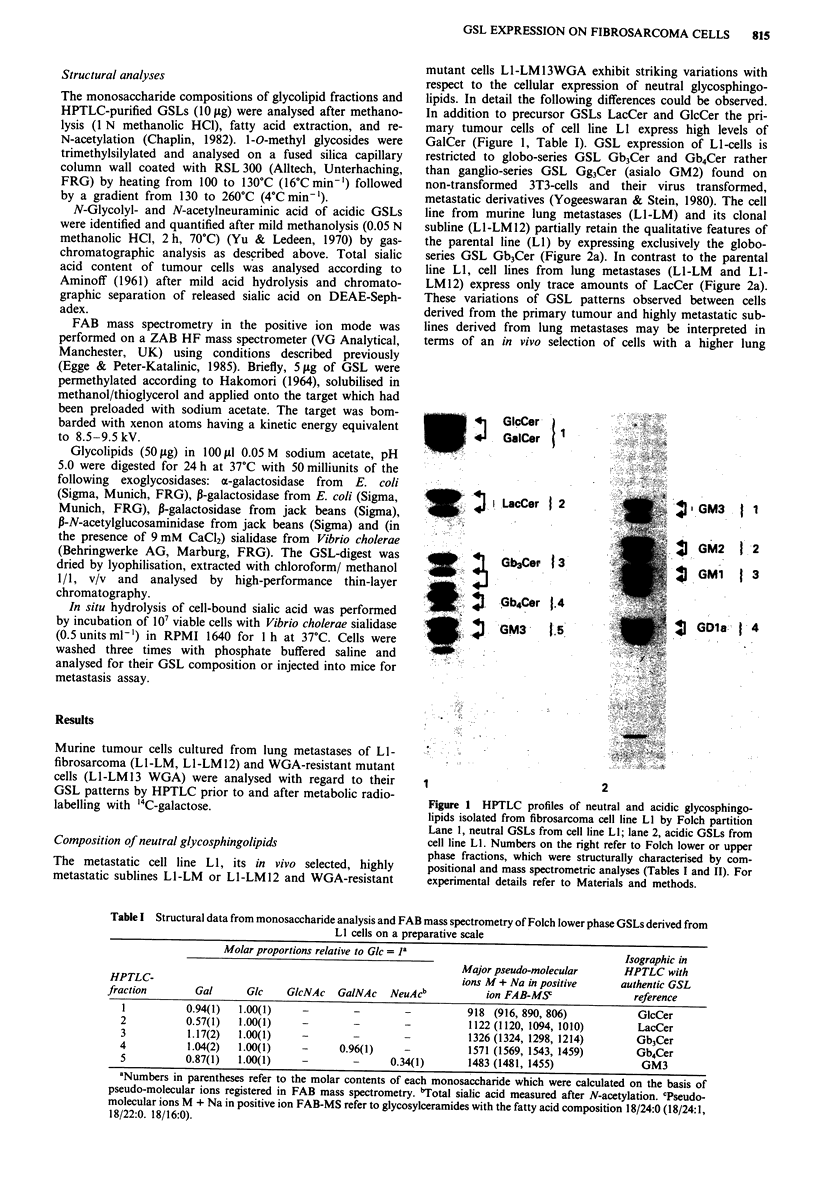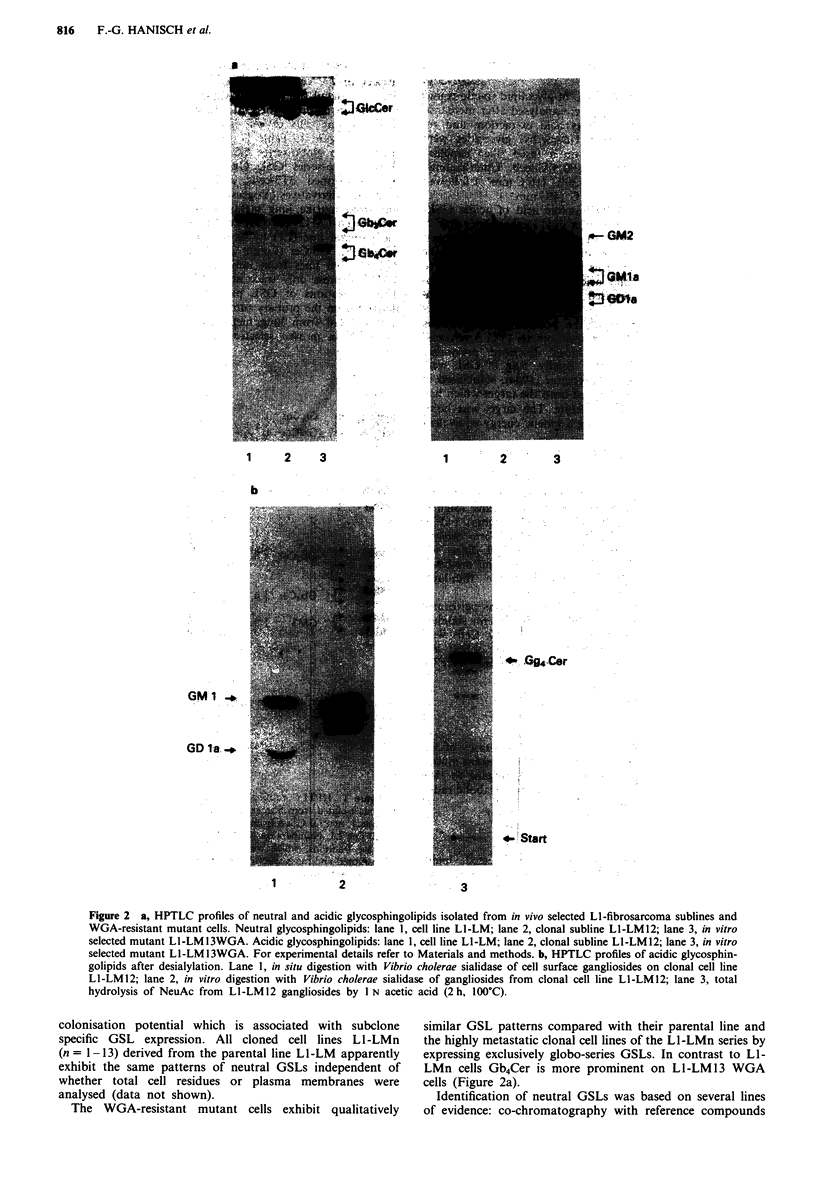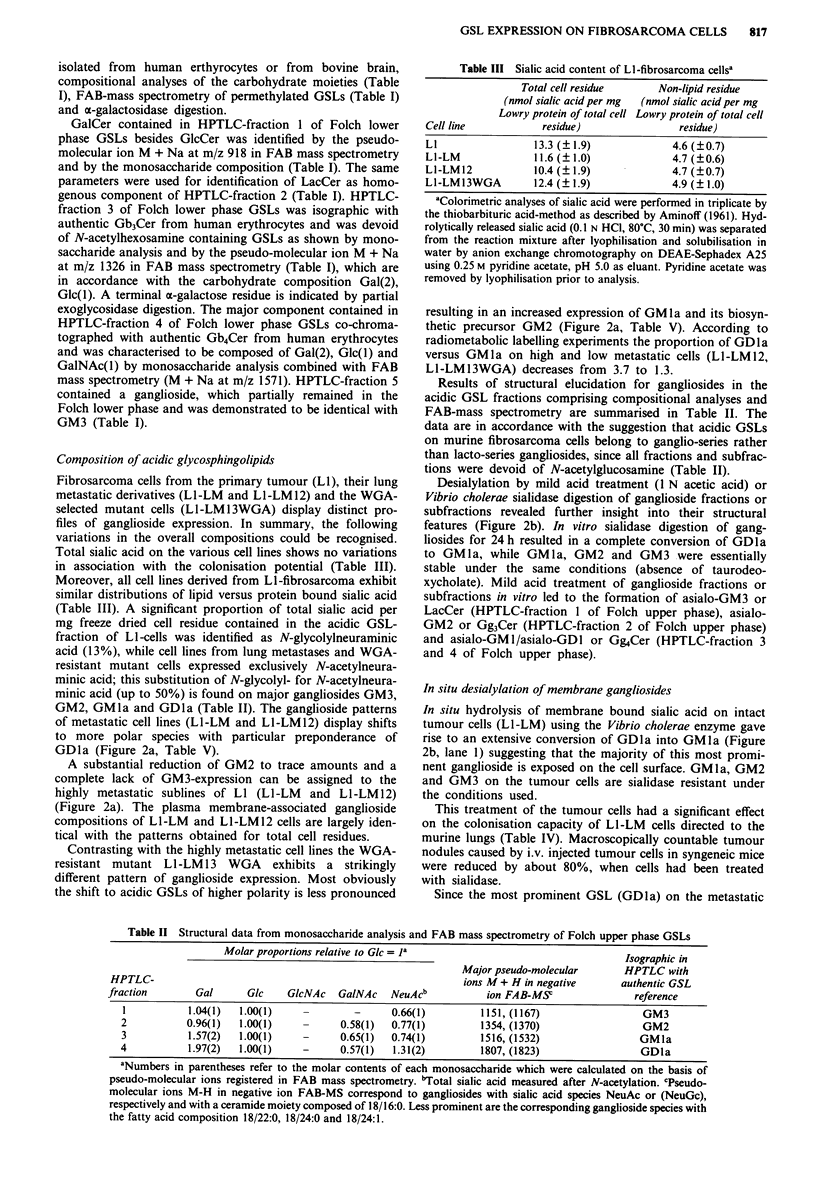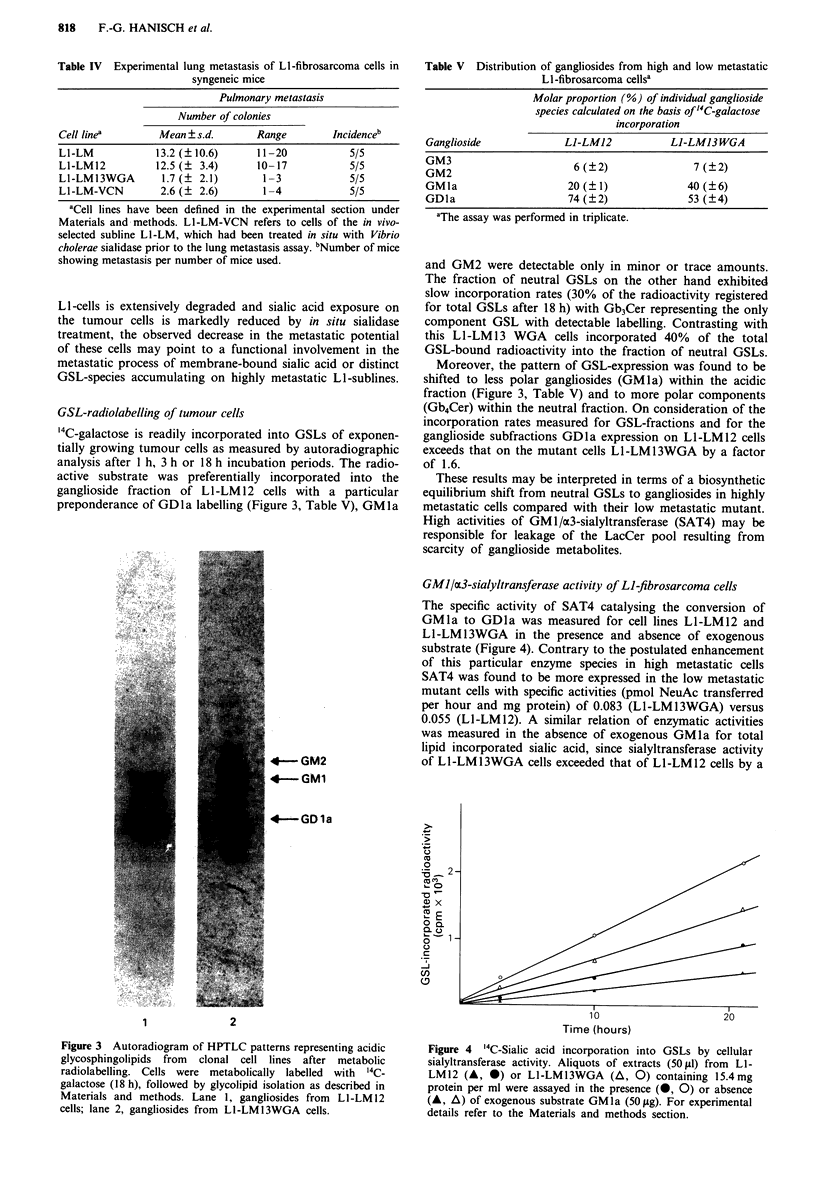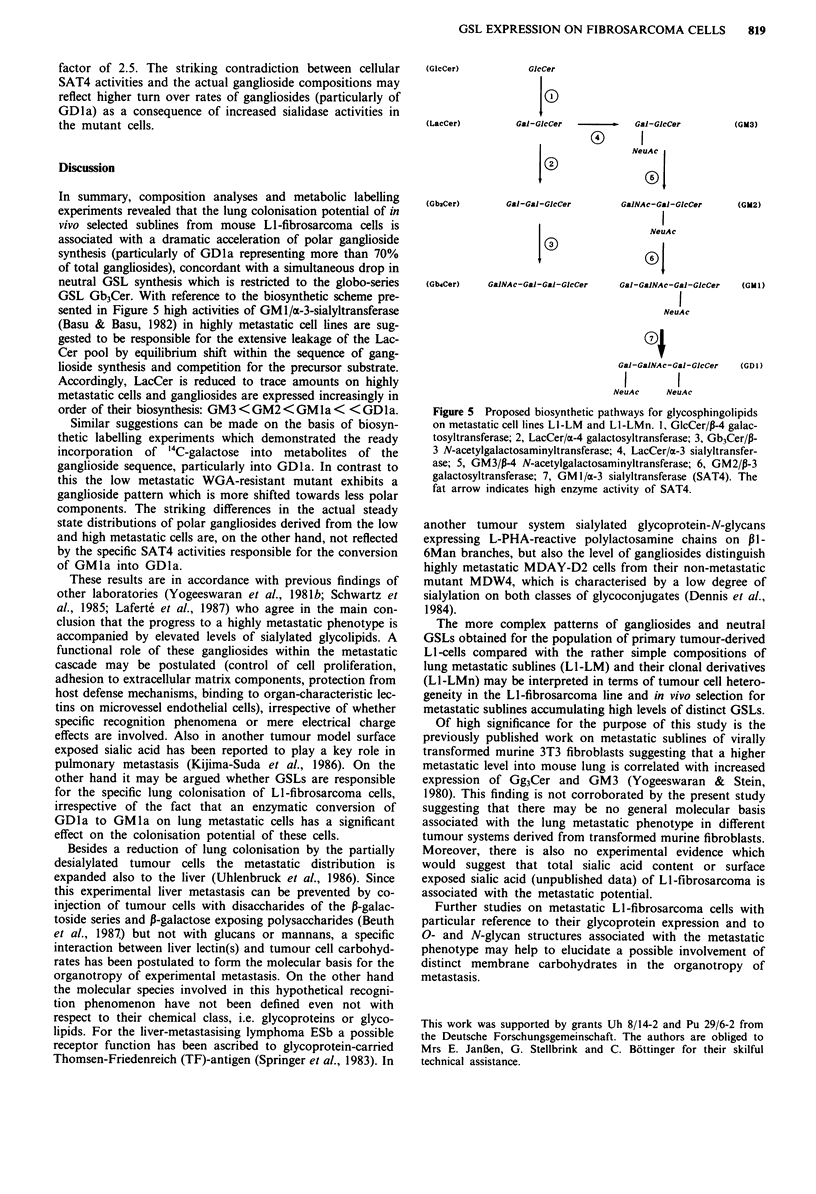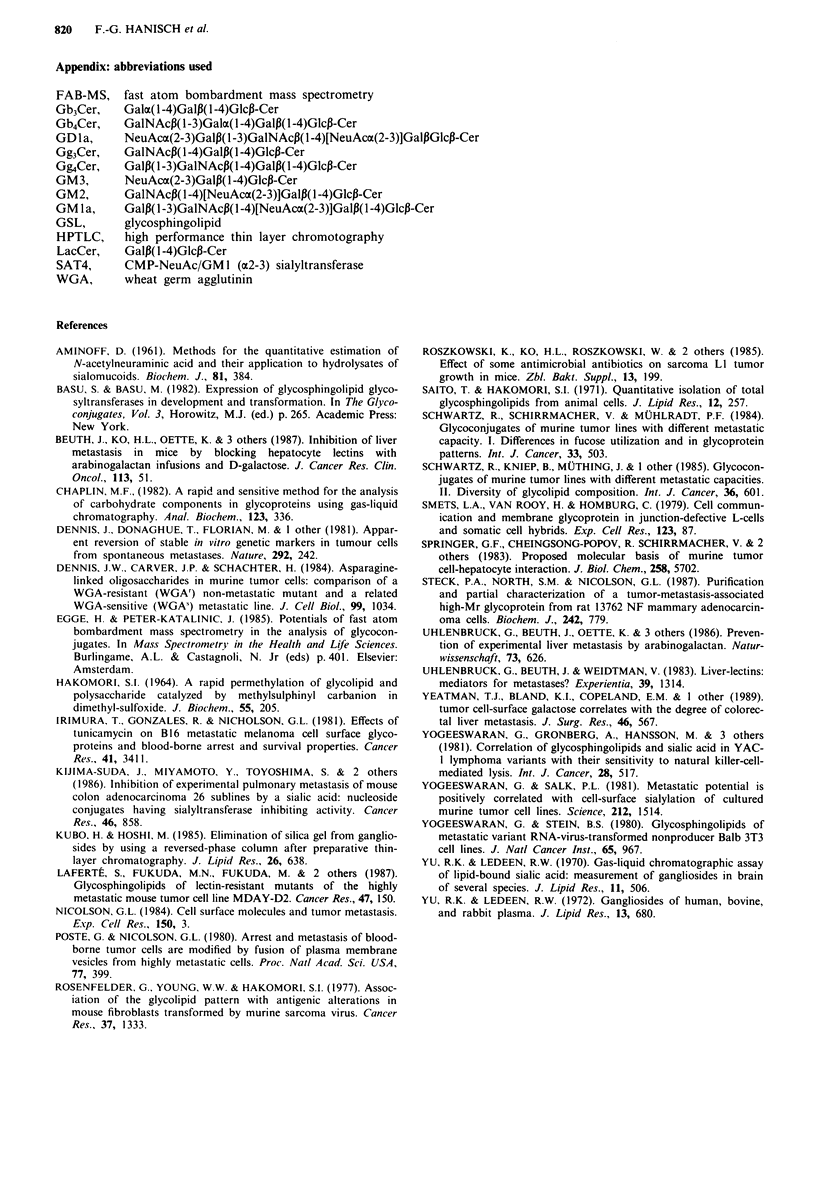# Glycosphingolipid expression on murine L1-fibrosarcoma cells: analysis of clonal in vivo and in vitro selected sublines with different lung colonisation potential.

**DOI:** 10.1038/bjc.1990.183

**Published:** 1990-06

**Authors:** F. G. Hanisch, J. Sölter, V. Jansen, A. Lochner, J. Peter-Katalinic, G. Uhlenbruck

**Affiliations:** Institute of Immunobiology, University Clinic of Cologne, Federal Republic of Germany.

## Abstract

**Images:**


*Br. J. Cancer* (1990), **61**, 813–820 © Macmillan Press Ltd., 1990

### Glycosphingolipid expression on murine L1-fibrosarcoma cells: analysis of clonal *in vivo* and *in vitro* selected sublines with different lung colonisation potential

F.-G. Hanisch, J. Sölter, V. Jansen, A. Lochner, J. Peter-Katalinic[1] & G. Uhlenbruck

*Institute of Immunobiology, University Clinic of Cologne, Kerpener Str. 15, D-5000 Cologne 41; and [1]Institute of Physiological Chemistry, University of Bonn, Nussallee 11, D-5300 Bonn, Federal Republic of Germany.*

**Summary** The patterns of acidic and neutral glycosphingolipids (GSLs) were examined in a syngeneic tumour system in Balb/c mice consisting of closely related cell lines with different colonisation potentials directed to the murine lungs (*in vivo* selected highly metastatic sublines of L1-fibrosarcoma cells and their WGA-resistant mutants with low metastatic potential). GSLs were analysed by high-performance thin-layer chromatography and structurally identified by fast atom bombardment mass spectrometry combined with compositional analyses and exo-glycosidase digestion. The results suggest that highly metastatic sublines L1-LM and L1-LM12 derived by *in vivo* selection from mouse fibrosarcoma cells (cell line L1) exhibit a drastic increase of polar ganglioside expression and a restriction to globo-series GSLs. Contrasting with this the low metastatic mutant cells (L1-LM13WGA) express a reduced portion of acidic GSLs and exhibit a shift to less polar ganglioside components. Total cellular and plasma membrane-integrated GSLs were demonstrated to exhibit largely identical patterns. Concomitant with a significant decrease in LacCer expression a substantial reduction of GM2 and a complete lack of GM3 expression can be assigned to the highly metastatic sublines of L1-cells. On the other hand, the more polar gangliosides GM1a and, to an even greater extent, GD1a (exceeding 70% of total gangliosides) accumulate on L1-LM and their clonal sublines. The shift to acidic GSLs of higher polarity is less pronounced on the low metastatic WGA-resistant mutant cells (L1-LM13WGA) showing a preponderance of GM1a. The portion of GD1a within the fractions of acidic GSLs does not correspond to the cellular activities of CMP-NeuAc/GM1 (α2–3) sialyltransferase measured for high and low metastatic cell variants. Total sialic acid content of the various cell lines differs, but is not associated with the metastatic potential. Gangliosides on L1-cells exhibit a significant substitution of *N*-glycolyl for *N*-acetylneuraminic acid (13%) compared to their metastatic sublines and to mutant cells (<1%). A conversion of surface exposed GD1a to GM1a on membranes of metastatic cells by *in situ* treatment with *Vibrio cholerae* sialidase is associated with a significant reduction of tumour cell colonisation directed to the murine lungs.

Transformation-related structural modifications of cell surface carbohydrates have been assumed to imply a series of functional aspects with regard to the social behaviour of the tumour cell within the metastatic cascade (Nicolson, 1984). Apart from the postulated role of membrane carbohydrates regarding tumour cell escape from the primary site (Smets *et al.*, 1979) and tumour cell survival of host defense mechanisms in the haemato-lymphoid system (Yogeeswaran *et al.*, 1981*a*; Irimura *et al.*, 1981) there is also evidence for tumour cell glycoproteins and glycolipids acting as mediators of metastatic organotropy by specific adhesion to organ lectins (Uhlenbruck *et al.*, 1983; Springer *et al.*, 1983; Yeatman *et al.*, 1989).

The involvement of membrane constituents has been demonstrated directly by transfer of the metastatic capacity by fusion of low metastatic cells with membrane vesicles from highly metastatic cells (Poste & Nicolson, 1980). Most of the relevant evidence is indirect suggesting that membrane constituents which are less expressed on non-metastatic cells or even absent may be responsible for the properties of metastatic tumour cells. Accordingly, high and low metastatic cells differ in cell surface sialic acid (Yogeeswaran *et al.*, 1981*b*) in glycolipid (Schwartz *et al.*, 1985; Laferté *et al.*, 1987) and in glycoprotein composition (Schwartz *et al.*, 1984; Steck *et al.*, 1987).

With regard to glycolipid expression on virally transformed murine fibroblasts alterations in gangliosides and neutral glycosphingolipids (GSLs) have been demonstrated (Rosenfelder *et al.*, 1977). Metastatic sublines of virally transformed murine Balb/c 3T3 fibroblasts have been reported to contain ganglioside GM3 (see Appendix for abbreviations) and $Gg_3Cer$ as predominant GSLs on the cell surface (Yogeeswaran & Stein, 1980).

Correspondence: F.-G. Hanisch.
Received 3 May 1989; and in revised form 15 December 1989.

Here we report on the GSL expression of highly metastatic murine Balb/c 3T3 cells, which have been derived from a spontaneous fibrosarcoma of the lung (Roszkowski *et al.*, 1985). A subpopulation of L1-fibrosarcoma cells is highly metastatic exclusively to the murine lungs, but has been demonstrated by experimental modification of membrane carbohydrates (sialidase treatment) to expand its metastatic distribution also to the liver (Uhlenbruck *et al.*, 1986). Since the latter process is inhibited by co-injection of polyvalent β-galactosides and free D-galactose, a specific interaction of terminal sugar residues on L1-plasma membrane glycoconjugates and organ-characteristic lectins of the murine liver has been suggested (Beuth *et al.*, 1987).

A similar 'homing' phenomenon in the organotropy of native L1-cells can also be postulated for murine lung colonisation. Since cell surface glycoconjugates of this tumour system have not been studied on a sound chemical basis, the present contribution is an attempt to define variations in the glycosphingolipid expression on L1-fibrosarcoma sublines colonising the lungs at different rates after i.v. inoculation.

### Materials and methods

### *Reference compounds*

Neutral glycospingolipids of the globo-series and sialyl-paragloboside were isolated from human erythrocyte membranes. Asialo-gangliosides of the ganglio-series were prepared from bovine brain gangliosides (Sigma, Munich, FRG) by treatment with 1 N acetic acid for 90 min at 100°C.

GM1a, GD1a and GT1 were purchased from Sigma (Munich, FRG).

### *Cell lines*

L1-fibrosarcoma cells (Institute of Oncology, Warsaw, Poland) derived from a tumour that had spontaneously arisen in the lung of Balb/c 3T3 had been maintained in Balb/c mice by implantation of L1-fibrosarcoma cells s.c. into the animals' limbs, inducing growth of a local solid tumour (Roszkowski et al., 1985).

Homogenates of L1 fibrosarcoma were used for s.c. inoculations into eight syngeneic Balb/c mice. Two weeks later the tumours were enucleated, minced with scissors and forced through a stainless steel mesh. The homogenate was filtered through a sterile cotton layer, washed and resuspended after lysis of erythrocytes into 100 ml growth medium for cell culture (RPMI 1640, containing 10% v/v fetal calf serum, 2 mM glutamine, 100 IU $ml^{-1}$ penicillin, 100 $\mu g\ ml^{-1}$ streptomycin and 2.5 $\mu g\ ml^{-1}$ amphotericin B). Cells were passaged at intervals of 2–3 days (cell line L1).

Sterile i.v. injections of $10^5$ viable L1 cells per 0.1 ml phosphate buffered saline into the tail veins of Balb/c mice induced multiple, non-confluent lung metastases within 14 days. The lungs were resected and the metastatic nodules were separated from the remaining tissue. Cells derived from lung metastases were prepared for cell culture as described for L1-fibrosarcoma cells. The cell line derived from murine lung metastases (L1-LM) was used for establishment of several cloned lines designated L1-LMn ($n = 1-13$). Cloning and recloning was performed by the limiting dilution method using trypsinised single cell suspensions diluted to 10, 50 and 100 cells $ml^{-1}$ (first cloning) and seeded in multi-well dishes. After 1 week in a humid $CO_2$ incubator colonies were isolated, grown up to $10^6$ cells $ml^{-1}$ and recloned by dilution to 10 cells $ml^{-1}$.

The relationship of cell lines examined in this study is as follows: primary tumour (L1-fibrosarcoma), to cell line L1, to lung metastases, to cell line L1-LM, to clonal cell lines L1-LMn ($n = 1-13$), to clonal WGA-resistant mutant L1-LM13 (WGA).

Lectin resistant mutant cells were selected by a procedure which has been described in detail (Dennis et al., 1981). Briefly, L1-LM cells and their clonal sublines with a high lung metastatic capacity were treated with ethyl methanesulphonate (360 $\mu g\ ml^{-1}$) for 18 h. The cells were washed and grown in the above described culture medium for 1 week prior to selection of WGA-resistant mutants. The cells were plated in growth medium plus WGA (10 $\mu g\ ml^{-1}$) at $10^4$ cells per well in 96-well microtest plates and positive wells were selected and cloned by limiting dilution. Lectin sensitivity of the tumour cells was tested by measuring $^3H$-thymidine incorporation rates into DNA in the presence of increasing concentrations of lectin. Cells were cultured at $10^4$ per well in 96-well microtest plates containing serial dilutions of lectin. After 2 days the cells were pulsed with 1 $\mu Ci$ of $^3H$-thymidine harvested 4 h later onto glass fibre discs and their incorporated radioactivity was counted in a liquid scintillation counter.

The tumour cells were regularly checked to be free of mycoplasma and to have retained their tumorigenic capacity. Experimental metastasis was performed by injecting 0.1 ml of viable tumour cells ($10^5$ cells $ml^{-1}$ PBS) into the tail veins of male, 2-month-old animals. After 20 days the animals were killed and the lungs were examined macroscopically for tumour colonies after staining with ink (Table IV). An estimation of the relative colonisation potential of the parental L1 and its *in vivo* selected subline L1-LM was based on experimental metastasis using 20 animals for each group and i.v. inoculation of $10^5$ tumour cells per animal. After two weeks one-third of the animals inoculated with L1-LM cells had died, while the remaining exhibited confluent, non-countable lung metastases. Under the same conditions, lungs from animals inoculated with L1 cells showed in the range of 50–100 countable tumour nodules and all animals had survived this time period.

### *Preparation of plasma membranes*

Briefly, cells were homogenised in 10 mM sodium phosphate, pH 7.4, containing 1 mM $MgCl_2$, 30 mM NaCl, 1 mM dithiotreitol, 0.005 mM phenylmethylsulphonyl fluoride, 0.02% $NaN_3$ and a few micrograms of DNAse and carefully layered over a 41% solution of sucrose in the homogenisation buffer. Ultracentrifugation was performed at 95,000 *g* for 1 h in a Beckman SW 27 swinging bucket rotor.

### *Metabolic radiolabelling*

Metabolic radiolabelling of exponentially growing cells ($10^6\ ml^{-1}$) was performed after resuspension in fresh RPMI 1640 medium containing 10% v/v FCS by incubation with 1 $\mu Ci\ ml^{-1}$ of D-$^{14}C$-galactose (specific activity: 50–60 mCi $mmol^{-1}$) (Amersham, Braunschweig, FRG). Aliquots of the cell suspension were withdrawn at various time intervals and the cells were washed twice with phosphate buffered saline prior to extraction of GSLs (see below) in the presence of 100 $\mu g$ aliquots of neutral and acidic carrier GSLs. Neutral and acidic fractions were analysed by HPTLC and the plates exposed to X-Omat X-ray film (Eastman Kodak, Heidelberg, FRG) for 1 week at $-20°C$. Following autoradiography the radioactive bands were scraped off from the plate, the GSLs were eluted from the silica gel with chloroform/methanol mixtures and counted for their incorporated radioactivity in a liquid scintillation counter.

### *Sialyltransferase assay*

Frozen cells were homogenised and solubilised in the cold by addition of 2 vol. of 0.1 M sodium cacodylate buffer (pH 6.5) containing 25% glycerol, 0.15 M NaCl and 2% Triton X-100. After vortexing for 2 min, the cells were centrifuged at 4°C for 15 min at 12,000 *g*. The supernatant was analysed for protein concentration using the Lowry procedure in the presence of SDS. Aliquots of the extracts (50 $\mu l$) containing 15.4 mg protein per ml were assayed for SAT4 activity using GM1a (50 $\mu g$) as substrate and 0.125 $\mu Ci$ of CMP-$^{14}C$-NeuAc. A control for endogenous activity was included for each cell line by omitting the exogenous substrate from the assay. Aliquots of 10 $\mu l$ of the assay mixture were withdrawn at various time intervals, heated for 2 min at 90°C and dried. The residue was extracted with 100 $\mu l$ of chloroform-methanol, 1/1 (v/v), and chromatographed on Whatman 3 paper using water as eluent. The GSLs remaining at the origin were eluted with chloroform-methanol-water, 10/5/1 (v/v) and analysed radiometrically for incorporated $^{14}C$-NeuAc in a $\beta$-scintillation counter.

### *Isolation of glycosphingolipids*

On an analytical scale, glycosphingolipids were isolated from approximately 0.2 ml of packed cells by sequential extraction with 5 vol. of chloroform/methanol 2/1, 1/1, 1/2, v/v. The combined extracts were concentrated to dryness and applied onto DEAE-Sephadex (acetate) for separation of neutral GSLs and gangliosides (Yu & Ledeen, 1972).

Gangliosides eluting with chloroform/methanol/0.8 M sodium acetate (30/60/8, v/v) were desalted on Sep Pak $C_{18}$ cartridges (Millipore) as described by Kubo and Hoshi (1985). Neutral GSLs were separated from other lipid components by acetylation and chromatography on Florisil (0.15–0.25 mm, Serva, Heidelberg, FRG) as described by Saito and Hakomori (1971). Large scale preparations were performed accordingly starting from 1–5 ml of packed cells and introducing a Folch-partition prior to DEAE-Sephadex chromatography.

### *Thin layer chromatography*

Glycosphingolipids corresponding to 10–20 $\mu g$ hexose were applied onto high-performance thin-layer plates (HPTLC silica gel, size 10 × 20 cm, Merck, Darmstadt, FRG) and chromatographed in chloroform/methanol/water 60/35/8, v/v (neutral fraction) or chloroform/methanol/0.02% $CaCl_2$, 60/35/8, v/v (acidic fraction). GSL bands were visualised by spraying with orcinol reagent.

*Structural analyses*

The monosaccharide compositions of glycolipid fractions and HPTLC-purified GSLs (10 μg) were analysed after methanolysis (1 N methanolic HCl), fatty acid extraction, and re-N-acetylation (Chaplin, 1982). 1-*O*-methyl glycosides were trimethylsilylated and analysed on a fused silica capillary column wall coated with RSL 300 (Alltech, Unterhaching, FRG) by heating from 100 to 130°C (16°C $min^{-1}$) followed by a gradient from 130 to 260°C (4°C $min^{-1}$).

*N*-Glycolyl- and *N*-acetylneuraminic acid of acidic GSLs were identified and quantified after mild methanolysis (0.05 N methanolic HCl, 2 h, 70°C) (Yu & Ledeen, 1970) by gas-chromatographic analysis as described above. Total sialic acid content of tumour cells was analysed according to Aminoff (1961) after mild acid hydrolysis and chromatographic separation of released sialic acid on DEAE-Sephadex.

FAB mass spectrometry in the positive ion mode was performed on a ZAB HF mass spectrometer (VG Analytical, Manchester, UK) using conditions described previously (Egge & Peter-Katalinic, 1985). Briefly, 5 μg of GSL were permethylated according to Hakomori (1964), solubilised in methanol/thioglycerol and applied onto the target which had been preloaded with sodium acetate. The target was bombarded with xenon atoms having a kinetic energy equivalent to 8.5–9.5 kV.

Glycolipids (50 μg) in 100 μl 0.05 M sodium acetate, pH 5.0 were digested for 24 h at 37°C with 50 milliunits of the following exoglycosidases: α-galactosidase from *E. coli* (Sigma, Munich, FRG), β-galactosidase from *E. coli* (Sigma, Munich, FRG), β-galactosidase from jack beans (Sigma), β-*N*-acetylglucosaminidase from jack beans (Sigma) and (in the presence of 9 mM $CaCl_2$) sialidase from *Vibrio cholerae* (Behringwerke AG, Marburg, FRG). The GSL-digest was dried by lyophilisation, extracted with chloroform/ methanol 1/1, v/v and analysed by high-performance thin-layer chromatography.

*In situ* hydrolysis of cell-bound sialic acid was performed by incubation of $10^7$ viable cells with *Vibrio cholerae* sialidase (0.5 units $ml^{-1}$) in RPMI 1640 for 1 h at 37°C. Cells were washed three times with phosphate buffered saline and analysed for their GSL composition or injected into mice for metastasis assay.

### Results

Murine tumour cells cultured from lung metastases of L1-fibrosarcoma (L1-LM, L1-LM12) and WGA-resistant mutant cells (L1-LM13 WGA) were analysed with regard to their GSL patterns by HPTLC prior to and after metabolic radio-labelling with $^{14}$C-galactose.

*Composition of neutral glycosphingolipids*

The metastatic cell line L1, its *in vivo* selected, highly metastatic sublines L1-LM or L1-LM12 and WGA-resistant mutant cells L1-LM13WGA exhibit striking variations with respect to the cellular expression of neutral glycosphingolipids. In detail the following differences could be observed. In addition to precursor GSLs LacCer and GlcCer the primary tumour cells of cell line L1 express high levels of GalCer (Figure 1, Table I). GSL expression of L1-cells is restricted to globo-series GSL $Gb_3$Cer and $Gb_4$Cer rather than ganglio-series GSL $Gg_3$Cer (asialo GM2) found on non-transformed 3T3-cells and their virus transformed, metastatic derivatives (Yogeeswaran & Stein, 1980). The cell line from murine lung metastases (L1-LM) and its clonal subline (L1-LM12) partially retain the qualitative features of the parental line (L1) by expressing exclusively the globo-series GSL $Gb_3$Cer (Figure 2a). In contrast to the parental line L1, cell lines from lung metastases (L1-LM and L1-LM12) express only trace amounts of LacCer (Figure 2a). These variations of GSL patterns observed between cells derived from the primary tumour and highly metastatic sublines derived from lung metastases may be interpreted in terms of an *in vivo* selection of cells with a higher lung

**Figure 1** HPTLC profiles of neutral and acidic glycosphingolipids isolated from fibrosarcoma cell line L1 by Folch partition Lane 1, neutral GSLs from cell line L1; lane 2, acidic GSLs from cell line L1. Numbers on the right refer to Folch lower or upper phase fractions, which were structurally characterised by compositional and mass spectrometric analyses (Tables I and II). For experimental details refer to Materials and methods.

**Table I** Structural data from monosaccharide analysis and FAB mass spectrometry of Folch lower phase GSLs derived from L1 cells on a preparative scale

| *HPTLC-fraction* | *Molar proportions relative to Glc = 1*[a] | | | | | *Major pseudo-molecular ions M + Na in positive ion FAB-MS*[c] | *Isographic in HPTLC with authentic GSL reference* |
|---|---|---|---|---|---|---|---|
| | *Gal* | *Glc* | *GlcNAc* | *GalNAc* | *NeuAc*[b] | | |
| 1 | 0.94(1) | 1.00(1) | – | – | – | 918 (916, 890, 806) | GlcCer |
| 2 | 0.57(1) | 1.00(1) | – | – | – | 1122 (1120, 1094, 1010) | LacCer |
| 3 | 1.17(2) | 1.00(1) | – | – | – | 1326 (1324, 1298, 1214) | $Gb_3$Cer |
| 4 | 1.04(2) | 1.00(1) | – | 0.96(1) | – | 1571 (1569, 1543, 1459) | $Gb_4$Cer |
| 5 | 0.87(1) | 1.00(1) | – | – | 0.34(1) | 1483 (1481, 1455) | GM3 |

[a]Numbers in parentheses refer to the molar contents of each monosaccharide which were calculated on the basis of pseudo-molecular ions registered in FAB mass spectrometry. [b]Total sialic acid measured after *N*-acetylation. [c]Pseudo-molecular ions M + Na in positive ion FAB-MS refer to glycosylceramides with the fatty acid composition 18/24:0 (18/24:1, 18/22:0. 18/16:0).

**Figure 2** **a,** HPTLC profiles of neutral and acidic glycosphingolipids isolated from *in vivo* selected L1-fibrosarcoma sublines and WGA-resistant mutant cells. Neutral glycosphingolipids: lane 1, cell line L1-LM; lane 2, clonal subline L1-LM12; lane 3, *in vitro* selected mutant L1-LM13WGA. Acidic glycosphingolipids: lane 1, cell line L1-LM; lane 2, clonal subline L1-LM12; lane 3, *in vitro* selected mutant L1-LM13WGA. For experimental details refer to Materials and methods. **b,** HPTLC profiles of acidic glycosphingolipids after desialylation. Lane 1, *in situ* digestion with *Vibrio cholerae* sialidase of cell surface gangliosides on clonal cell line L1-LM12; lane 2, *in vitro* digestion with *Vibrio cholerae* sialidase of gangliosides from clonal cell line L1-LM12; lane 3, total hydrolysis of NeuAc from L1-LM12 gangliosides by 1 N acetic acid (2 h, 100°C).

colonisation potential which is associated with subclone specific GSL expression. All cloned cell lines L1-LMn ($n = 1-13$) derived from the parental line L1-LM apparently exhibit the same patterns of neutral GSLs independent of whether total cell residues or plasma membranes were analysed (data not shown).

The WGA-resistant mutant cells exhibit qualitatively similar GSL patterns compared with their parental line and the highly metastatic clonal cell lines of the L1-LMn series by expressing exclusively globo-series GSLs. In contrast to L1-LMn cells $Gb_4Cer$ is more prominent on L1-LM13 WGA cells (Figure 2a).

Identification of neutral GSLs was based on several lines of evidence: co-chromatography with reference compounds isolated from human erthyrocytes or from bovine brain, compositional analyses of the carbohydrate moieties (Table I), FAB-mass spectrometry of permethylated GSLs (Table I) and α-galactosidase digestion.

GalCer contained in HPTLC-fraction 1 of Folch lower phase GSLs besides GlcCer was identified by the pseudo-molecular ion M + Na at m/z 918 in FAB mass spectrometry and by the monosaccharide composition (Table I). The same parameters were used for identification of LacCer as homogenous component of HPTLC-fraction 2 (Table I). HPTLC-fraction 3 of Folch lower phase GSLs was isographic with authentic $Gb_3Cer$ from human erythrocytes and was devoid of N-acetylhexosamine containing GSLs as shown by monosaccharide analysis and by the pseudo-molecular ion M + Na at m/z 1326 in FAB mass spectrometry (Table I), which are in accordance with the carbohydrate composition Gal(2), Glc(1). A terminal α-galactose residue is indicated by partial exoglycosidase digestion. The major component contained in HPTLC-fraction 4 of Folch lower phase GSLs co-chromatographed with authentic $Gb_4Cer$ from human erythrocytes and was characterised to be composed of Gal(2), Glc(1) and GalNAc(1) by monosaccharide analysis combined with FAB mass spectrometry (M + Na at m/z 1571). HPTLC-fraction 5 contained a ganglioside, which partially remained in the Folch lower phase and was demonstrated to be identical with GM3 (Table I).

*Composition of acidic glycosphingolipids*

Fibrosarcoma cells from the primary tumour (L1), their lung metastatic derivatives (L1-LM and L1-LM12) and the WGA-selected mutant cells (L1-LM13WGA) display distinct profiles of ganglioside expression. In summary, the following variations in the overall compositions could be recognised. Total sialic acid on the various cell lines shows no variations in association with the colonisation potential (Table III). Moreover, all cell lines derived from L1-fibrosarcoma exhibit similar distributions of lipid versus protein bound sialic acid (Table III). A significant proportion of total sialic acid per mg freeze dried cell residue contained in the acidic GSL-fraction of L1-cells was identified as N-glycolylneuraminic acid (13%), while cell lines from lung metastases and WGA-resistant mutant cells expressed exclusively N-acetylneuraminic acid; this substitution of N-glycolyl- for N-acetylneuraminic acid (up to 50%) is found on major gangliosides GM3, GM2, GM1a and GD1a (Table II). The ganglioside patterns of metastatic cell lines (L1-LM and L1-LM12) display shifts to more polar species with particular preponderance of GD1a (Figure 2a, Table V).

A substantial reduction of GM2 to trace amounts and a complete lack of GM3-expression can be assigned to the highly metastatic sublines of L1 (L1-LM and L1-LM12) (Figure 2a). The plasma membrane-associated ganglioside compositions of L1-LM and L1-LM12 cells are largely identical with the patterns obtained for total cell residues.

Contrasting with the highly metastatic cell lines the WGA-resistant mutant L1-LM13 WGA exhibits a strikingly different pattern of ganglioside expression. Most obviously the shift to acidic GSLs of higher polarity is less pronounced

**Table III** Sialic acid content of L1-fibrosarcoma cells[a]

| *Cell line* | *Total cell residue (nmol sialic acid per mg Lowry protein of total cell residue)* | *Non-lipid residue (nmol sialic acid per mg Lowry protein of total cell residue)* |
|---|---|---|
| L1 | 13.3 (±1.9) | 4.6 (±0.7) |
| L1-LM | 11.6 (±1.0) | 4.7 (±0.6) |
| L1-LM12 | 10.4 (±1.9) | 4.7 (±0.7) |
| L1-LM13WGA | 12.4 (±1.9) | 4.9 (±1.0) |

[a]Colorimetric analyses of sialic acid were performed in triplicate by the thiobarbituric acid-method as described by Aminoff (1961). Hydrolytically released sialic acid (0.1 N HCl, 80°C, 30 min) was separated from the reaction mixture after lyophilisation and solubilisation in water by anion exchange chromotography on DEAE-Sephadex A25 using 0.25 M pyridine acetate, pH 5.0 as eluant. Pyridine acetate was removed by lyophilisation prior to analysis.

resulting in an increased expression of GM1a and its biosynthetic precursor GM2 (Figure 2a, Table V). According to radiometabolic labelling experiments the proportion of GD1a versus GM1a on high and low metastatic cells (L1-LM12, L1-LM13WGA) decreases from 3.7 to 1.3.

Results of structural elucidation for gangliosides in the acidic GSL fractions comprising compositional analyses and FAB-mass spectrometry are summarised in Table II. The data are in accordance with the suggestion that acidic GSLs on murine fibrosarcoma cells belong to ganglio-series rather than lacto-series gangliosides, since all fractions and subfractions were devoid of N-acetylglucosamine (Table II).

Desialylation by mild acid treatment (1 N acetic acid) or *Vibrio cholerae* sialidase digestion of ganglioside fractions or subfractions revealed further insight into their structural features (Figure 2b). *In vitro* sialidase digestion of gangliosides for 24 h resulted in a complete conversion of GD1a to GM1a, while GM1a, GM2 and GM3 were essentially stable under the same conditions (absence of taurodeoxycholate). Mild acid treatment of ganglioside fractions or subfractions *in vitro* led to the formation of asialo-GM3 or LacCer (HPTLC-fraction 1 of Folch upper phase), asialo-GM2 or $Gg_3Cer$ (HPTLC-fraction 2 of Folch upper phase) and asialo-GM1/asialo-GD1 or $Gg_4Cer$ (HPTLC-fraction 3 and 4 of Folch upper phase).

*In situ desialylation of membrane gangliosides*

*In situ* hydrolysis of membrane bound sialic acid on intact tumour cells (L1-LM) using the *Vibrio cholerae* enzyme gave rise to an extensive conversion of GD1a into GM1a (Figure 2b, lane 1) suggesting that the majority of this most prominent ganglioside is exposed on the cell surface. GM1a, GM2 and GM3 on the tumour cells are sialidase resistant under the conditions used.

This treatment of the tumour cells had a significant effect on the colonisation capacity of L1-LM cells directed to the murine lungs (Table IV). Macroscopically countable tumour nodules caused by i.v. injected tumour cells in syngeneic mice were reduced by about 80%, when cells had been treated with sialidase.

Since the most prominent GSL (GD1a) on the metastatic

**Table II** Structural data from monosaccharide analysis and FAB mass spectrometry of Folch upper phase GSLs

| *HPTLC-fraction* | *Molar proportions relative to Glc = 1[a]* Gal | Glc | GlcNAc | GalNAc | NeuAc[b] | *Major pseudo-molecular ions M + H in negative ion FAB-MS[c]* | *Isographic in HPTLC with authentic GSL reference* |
|---|---|---|---|---|---|---|---|
| 1 | 1.04(1) | 1.00(1) | – | – | 0.66(1) | 1151, (1167) | GM3 |
| 2 | 0.96(1) | 1.00(1) | – | 0.58(1) | 0.77(1) | 1354, (1370) | GM2 |
| 3 | 1.57(2) | 1.00(1) | – | 0.65(1) | 0.74(1) | 1516, (1532) | GM1a |
| 4 | 1.97(2) | 1.00(1) | – | 0.57(1) | 1.31(2) | 1807, (1823) | GD1a |

[a]Numbers in parentheses refer to the molar contents of each monosaccharide which were calculated on the basis of pseudo-molecular ions registered in FAB mass spectrometry. [b]Total sialic acid measured after N-acetylation. [c]Pseudo-molecular ions M-H in negative ion FAB-MS correspond to gangliosides with sialic acid species NeuAc or (NeuGc), respectively and with a ceramide moiety composed of 18/16:0. Less prominent are the corresponding ganglioside species with the fatty acid composition 18/22:0, 18/24:0 and 18/24:1.

**Table IV** Experimental lung metastasis of L1-fibrosarcoma cells in syngeneic mice

| Cell line[a] | *Pulmonary metastasis* Number of colonies Mean ± s.d. | Range | Incidence[b] |
|---|---|---|---|
| L1-LM | 13.2 (±10.6) | 11–20 | 5/5 |
| L1-LM12 | 12.5 (± 3.4) | 10–17 | 5/5 |
| L1-LM13WGA | 1.7 (± 2.1) | 1–3 | 5/5 |
| L1-LM-VCN | 2.6 (± 2.6) | 1–4 | 5/5 |

[a]Cell lines have been defined in the experimental section under Materials and methods. L1-LM-VCN refers to cells of the *in vivo*-selected subline L1-LM, which had been treated *in situ* with *Vibrio cholerae* sialidase prior to the lung metastasis assay. [b]Number of mice showing metastasis per number of mice used.

L1-cells is extensively degraded and sialic acid exposure on the tumour cells is markedly reduced by *in situ* sialidase treatment, the observed decrease in the metastatic potential of these cells may point to a functional involvement in the metastatic process of membrane-bound sialic acid or distinct GSL-species accumulating on highly metastatic L1-sublines.

### *GSL-radiolabelling of tumour cells*

$^{14}$C-galactose is readily incorporated into GSLs of exponentially growing tumour cells as measured by autoradiographic analysis after 1 h, 3 h or 18 h incubation periods. The radioactive substrate was preferentially incorporated into the ganglioside fraction of L1-LM12 cells with a particular preponderance of GD1a labelling (Figure 3, Table V), GM1a

**Figure 3** Autoradiogram of HPTLC patterns representing acidic glycosphingolipids from clonal cell lines after metabolic radiolabelling. Cells were metabolically labelled with $^{14}$C-galactose (18 h), followed by glycolipid isolation as described in Materials and methods. Lane 1, gangliosides from L1-LM12 cells; lane 2, gangliosides from L1-LM13WGA cells.

**Table V** Distribution of gangliosides from high and low metastatic L1-fibrosarcoma cells[a]

| Ganglioside | *Molar proportion (%) of individual ganglioside species calculated on the basis of $^{14}$C-galactose incorporation* L1-LM12 | L1-LM13WGA |
|---|---|---|
| GM3 GM2 | 6 (±2) | 7 (±2) |
| GM1a | 20 (±1) | 40 (±6) |
| GD1a | 74 (±2) | 53 (±4) |

[a]The assay was performed in triplicate.

and GM2 were detectable only in minor or trace amounts. The fraction of neutral GSLs on the other hand exhibited slow incorporation rates (30% of the radioactivity registered for total GSLs after 18 h) with $Gb_3Cer$ representing the only component GSL with detectable labelling. Contrasting with this L1-LM13 WGA cells incorporated 40% of the total GSL-bound radioactivity into the fraction of neutral GSLs.

Moreover, the pattern of GSL-expression was found to be shifted to less polar gangliosides (GM1a) within the acidic fraction (Figure 3, Table V) and to more polar components ($Gb_4Cer$) within the neutral fraction. On consideration of the incorporation rates measured for GSL-fractions and for the ganglioside subfractions GD1a expression on L1-LM12 cells exceeds that on the mutant cells L1-LM13WGA by a factor of 1.6.

These results may be interpreted in terms of a biosynthetic equilibrium shift from neutral GSLs to gangliosides in highly metastatic cells compared with their low metastatic mutant. High activities of GM1/α3-sialyltransferase (SAT4) may be responsible for leakage of the LacCer pool resulting from scarcity of ganglioside metabolites.

### *GM1/α3-sialyltransferase activity of L1-fibrosarcoma cells*

The specific activity of SAT4 catalysing the conversion of GM1a to GD1a was measured for cell lines L1-LM12 and L1-LM13WGA in the presence and absence of exogenous substrate (Figure 4). Contrary to the postulated enhancement of this particular enzyme species in high metastatic cells SAT4 was found to be more expressed in the low metastatic mutant cells with specific activities (pmol NeuAc transferred per hour and mg protein) of 0.083 (L1-LM13WGA) versus 0.055 (L1-LM12). A similar relation of enzymatic activities was measured in the absence of exogenous GM1a for total lipid incorporated sialic acid, since sialyltransferase activity of L1-LM13WGA cells exceeded that of L1-LM12 cells by a

**Figure 4** $^{14}$C-Sialic acid incorporation into GSLs by cellular sialyltransferase activity. Aliquots of extracts (50 μl) from L1-LM12 (▲, ●) or L1-LM13WGA (Δ, O) containing 15.4 mg protein per ml were assayed in the presence (●, O) or absence (▲, Δ) of exogenous substrate GM1a (50 μg). For experimental details refer to the Materials and methods section.

factor of 2.5. The striking contradiction between cellular SAT4 activities and the actual ganglioside compositions may reflect higher turn over rates of gangliosides (particularly of GD1a) as a consequence of increased sialidase activities in the mutant cells.

### Discussion

In summary, composition analyses and metabolic labelling experiments revealed that the lung colonisation potential of *in vivo* selected sublines from mouse L1-fibrosarcoma cells is associated with a dramatic acceleration of polar ganglioside synthesis (particularly of GD1a representing more than 70% of total gangliosides), concordant with a simultaneous drop in neutral GSL synthesis which is restricted to the globo-series GSL $Gb_3Cer$. With reference to the biosynthetic scheme presented in Figure 5 high activities of GM1/α-3-sialyltransferase (Basu & Basu, 1982) in highly metastatic cell lines are suggested to be responsible for the extensive leakage of the LacCer pool by equilibrium shift within the sequence of ganglioside synthesis and competition for the precursor substrate. Accordingly, LacCer is reduced to trace amounts on highly metastatic cells and gangliosides are expressed increasingly in order of their biosynthesis: GM3 < GM2 < GM1a < < GD1a.

Similar suggestions can be made on the basis of biosynthetic labelling experiments which demonstrated the ready incorporation of $^{14}C$-galactose into metabolites of the ganglioside sequence, particularly into GD1a. In contrast to this the low metastatic WGA-resistant mutant exhibits a ganglioside pattern which is more shifted towards less polar components. The striking differences in the actual steady state distributions of polar gangliosides derived from the low and high metastatic cells are, on the other hand, not reflected by the specific SAT4 activities responsible for the conversion of GM1a into GD1a.

These results are in accordance with previous findings of other laboratories (Yogeeswaran *et al.*, 1981*b*; Schwartz *et al.*, 1985; Laferté *et al.*, 1987) who agree in the main conclusion that the progress to a highly metastatic phenotype is accompanied by elevated levels of sialylated glycolipids. A functional role of these gangliosides within the metastatic cascade may be postulated (control of cell proliferation, adhesion to extracellular matrix components, protection from host defense mechanisms, binding to organ-characteristic lectins on microvessel endothelial cells), irrespective of whether specific recognition phenomena or mere electrical charge effects are involved. Also in another tumour model surface exposed sialic acid has been reported to play a key role in pulmonary metastasis (Kijima-Suda *et al.*, 1986). On the other hand it may be argued whether GSLs are responsible for the specific lung colonisation of L1-fibrosarcoma cells, irrespective of the fact that an enzymatic conversion of GD1a to GM1a on lung metastatic cells has a significant effect on the colonisation potential of these cells.

Besides a reduction of lung colonisation by the partially desialylated tumour cells the metastatic distribution is expanded also to the liver (Uhlenbruck *et al.*, 1986). Since this experimental liver metastasis can be prevented by co-injection of tumour cells with disaccharides of the β-galactoside series and β-galactose exposing polysaccharides (Beuth *et al.*, 1987) but not with glucans or mannans, a specific interaction between liver lectin(s) and tumour cell carbohydrates has been postulated to form the molecular basis for the organotropy of experimental metastasis. On the other hand the molecular species involved in this hypothetical recognition phenomenon have not been defined even not with respect to their chemical class, i.e. glycoproteins or glycolipids. For the liver-metastasising lymphoma ESb a possible receptor function has been ascribed to glycoprotein-carried Thomsen-Friedenreich (TF)-antigen (Springer *et al.*, 1983). In another tumour system sialylated glycoprotein-*N*-glycans expressing L-PHA-reactive polylactosamine chains on β1-6Man branches, but also the level of gangliosides distinguish highly metastatic MDAY-D2 cells from their non-metastatic mutant MDW4, which is characterised by a low degree of sialylation on both classes of glycoconjugates (Dennis *et al.*, 1984).

**Figure 5** Proposed biosynthetic pathways for glycosphingolipids on metastatic cell lines L1-LM and L1-LMn. 1, GlcCer/β-4 galactosyltransferase; 2, LacCer/α-4 galactosyltransferase; 3, $Gb_3Cer$/β-3 *N*-acetylgalactosaminyltransferase; 4, LacCer/α-3 sialyltransferase; 5, GM3/β-4 *N*-acetylgalactosaminyltransferase; 6, GM2/β-3 galactosyltransferase; 7, GM1/α-3 sialyltransferase (SAT4). The fat arrow indicates high enzyme activity of SAT4.

The more complex patterns of gangliosides and neutral GSLs obtained for the population of primary tumour-derived L1-cells compared with the rather simple compositions of lung metastatic sublines (L1-LM) and their clonal derivatives (L1-LMn) may be interpreted in terms of tumour cell heterogeneity in the L1-fibrosarcoma line and *in vivo* selection for metastatic sublines accumulating high levels of distinct GSLs.

Of high significance for the purpose of this study is the previously published work on metastatic sublines of virally transformed murine 3T3 fibroblasts suggesting that a higher metastatic level into mouse lung is correlated with increased expression of $Gg_3Cer$ and GM3 (Yogeeswaran & Stein, 1980). This finding is not corroborated by the present study suggesting that there may be no general molecular basis associated with the lung metastatic phenotype in different tumour systems derived from transformed murine fibroblasts. Moreover, there is also no experimental evidence which would suggest that total sialic acid content or surface exposed sialic acid (unpublished data) of L1-fibrosarcoma is associated with the metastatic potential.

Further studies on metastatic L1-fibrosarcoma cells with particular reference to their glycoprotein expression and to *O*- and *N*-glycan structures associated with the metastatic phenotype may help to elucidate a possible involvement of distinct membrane carbohydrates in the organotropy of metastasis.

This work was supported by grants Uh 8/14-2 and Pu 29/6-2 from the Deutsche Forschungsgemeinschaft. The authors are obliged to Mrs E. Janßen, G. Stellbrink and C. Böttinger for their skilful technical assistance.

### Appendix: abbreviations used

| | |
|---|---|
| FAB-MS, | fast atom bombardment mass spectrometry |
| $Gb_3Cer$, | Galα(1-4)Galβ(1-4)Glcβ-Cer |
| $Gb_4Cer$, | GalNAcβ(1-3)Galα(1-4)Galβ(1-4)Glcβ-Cer |
| GD1a, | NeuAcα(2-3)Galβ(1-3)GalNAcβ(1-4)[NeuAcα(2-3)]GalβGlcβ-Cer |
| $Gg_3Cer$, | GalNAcβ(1-4)Galβ(1-4)Glcβ-Cer |
| $Gg_4Cer$, | Galβ(1-3)GalNAcβ(1-4)Galβ(1-4)Glcβ-Cer |
| GM3, | NeuAcα(2-3)Galβ(1-4)Glcβ-Cer |
| GM2, | GalNAcβ(1-4)[NeuAcα(2-3)]Galβ(1-4)Glcβ-Cer |
| GM1a, | Galβ(1-3)GalNAcβ(1-4)[NeuAcα(2-3)]Galβ(1-4)Glcβ-Cer |
| GSL, | glycosphingolipid |
| HPTLC, | high performance thin layer chromotography |
| LacCer, | Galβ(1-4)Glcβ-Cer |
| SAT4, | CMP-NeuAc/GM1 (α2-3) sialyltransferase |
| WGA, | wheat germ agglutinin |

### References

AMINOFF, D. (1961). Methods for the quantitative estimation of *N*-acetylneuraminic acid and their application to hydrolysates of sialomucoids. *Biochem. J.*, **81**, 384.

BASU, S. & BASU, M. (1982). Expression of glycosphingolipid glycosyltransferases in development and transformation. In *The Glycoconjugates, Vol. 3*, Horowitz, M.J. (ed.) p. 265. Academic Press: New York.

BEUTH, J., KO, H.L., OETTE, K. & 3 others (1987). Inhibition of liver metastasis in mice by blocking hepatocyte lectins with arabinogalactan infusions and D-galactose. *J. Cancer Res. Clin. Oncol.*, **113**, 51.

CHAPLIN, M.F., (1982). A rapid and sensitive method for the analysis of carbohydrate components in glycoproteins using gas-liquid chromatography. *Anal. Biochem.*, **123**, 336.

DENNIS, J., DONAGHUE, T., FLORIAN, M. & 1 other (1981). Apparent reversion of stable *in vitro* genetic markers in tumour cells from spontaneous metastases. *Nature*, **292**, 242.

DENNIS, J.W., CARVER, J.P. & SCHACHTER, H. (1984). Asparagine-linked oligosaccharides in murine tumor cells: comparison of a WGA-resistant ($WGA^r$) non-metastatic mutant and a related WGA-sensitive ($WGA^s$) metastatic line. *J. Cell Biol.*, **99**, 1034.

EGGE, H. & PETER-KATALINIC, J. (1985). Potentials of fast atom bombardment mass spectrometry in the analysis of glycoconjugates. In *Mass Spectrometry in the Health and Life Sciences*. Burlingame, A.L. & Castagnoli, N. Jr (eds) p. 401. Elsevier: Amsterdam.

HAKOMORI, S.I. (1964). A rapid permethylation of glycolipid and polysaccharide catalyzed by methylsulphinyl carbanion in dimethyl-sulfoxide. *J. Biochem.*, **55**, 205.

IRIMURA, T., GONZALES, R. & NICHOLSON, G.L. (1981). Effects of tunicamycin on B16 metastatic melanoma cell surface glycoproteins and blood-borne arrest and survival properties. *Cancer Res.*, **41**, 3411.

KIJIMA-SUDA, J., MIYAMOTO, Y., TOYOSHIMA, S. & 2 others (1986). Inhibition of experimental pulmonary metastasis of mouse colon adenocarcinoma 26 sublines by a sialic acid: nucleoside conjugates having sialyltransferase inhibiting activity. *Cancer Res.*, **46**, 858.

KUBO, H. & HOSHI, M. (1985). Elimination of silica gel from gangliosides by using a reversed-phase column after preparative thin-layer chromatography. *J. Lipid Res.*, **26**, 638.

LAFERTÉ, S., FUKUDA, M.N., FUKUDA, M. & 2 others (1987). Glycosphingolipids of lectin-resistant mutants of the highly metastatic mouse tumor cell line MDAY-D2. *Cancer Res.*, **47**, 150.

NICOLSON, G.L. (1984). Cell surface molecules and tumor metastasis. *Exp. Cell Res.*, **150**, 3.

POSTE, G. & NICOLSON, G.L. (1980). Arrest and metastasis of blood-borne tumor cells are modified by fusion of plasma membrane vesicles from highly metastatic cells. *Proc. Natl Acad. Sci. USA*, **77**, 399.

ROSENFELDER, G., YOUNG, W.W. & HAKOMORI, S.I. (1977). Association of the glycolipid pattern with antigenic alterations in mouse fibroblasts transformed by murine sarcoma virus. *Cancer Res.*, **37**, 1333.

ROSZKOWSKI, K., KO, H.L., ROSZKOWSKI, W. & 2 others (1985). Effect of some antimicrobial antibiotics on sarcoma L1 tumor growth in mice. *Zbl. Bakt. Suppl.*, **13**, 199.

SAITO, T. & HAKOMORI, S.I. (1971). Quantitative isolation of total glycosphingolipids from animal cells. *J. Lipid Res.*, **12**, 257.

SCHWARTZ, R., SCHIRRMACHER, V. & MÜHLRADT, P.F. (1984). Glycoconjugates of murine tumor lines with different metastatic capacity. I. Differences in fucose utilization and in glycoprotein patterns. *Int. J. Cancer*, **33**, 503.

SCHWARTZ, R., KNIEP, B., MÜTHING, J. & 1 other (1985). Glycoconjugates of murine tumor lines with different metastatic capacities. II. Diversity of glycolipid composition. *Int. J. Cancer*, **36**, 601.

SMETS, L.A., VAN ROOY, H. & HOMBURG, C. (1979). Cell communication and membrane glycoprotein in junction-defective L-cells and somatic cell hybrids. *Exp. Cell Res.*, **123**, 87.

SPRINGER, G.F., CHEINGSONG-POPOV, R. SCHIRRMACHER, V. & 2 others (1983). Proposed molecular basis of murine tumor cell-hepatocyte interaction. *J. Biol. Chem.*, **258**, 5702.

STECK, P.A., NORTH, S.M. & NICOLSON, G.L. (1987). Purification and partial characterization of a tumor-metastasis-associated high-Mr glycoprotein from rat 13762 NF mammary adenocarcinoma cells. *Biochem. J.*, **242**, 779.

UHLENBRUCK, G., BEUTH, J., OETTE, K. & 3 others (1986). Prevention of experimental liver metastasis by arabinogalactan. *Naturwissenschaft*, **73**, 626.

UHLENBRUCK, G., BEUTH, J. & WEIDTMAN, V. (1983). Liver-lectins: mediators for metastases? *Experientia*, **39**, 1314.

YEATMAN, T.J., BLAND, K.I., COPELAND, E.M. & 1 other (1989). tumor cell-surface galactose correlates with the degree of colorectal liver metastasis. *J. Surg. Res.*, **46**, 567.

YOGEESWARAN, G., GRONBERG, A., HANSSON, M. & 3 others (1981). Correlation of glycosphingolipids and sialic acid in YAC-1 lymphoma variants with their sensitivity to natural killer-cell-mediated lysis. *Int. J. Cancer*, **28**, 517.

YOGEESWARAN, G. & SALK, P.L. (1981). Metastatic potential is positively correlated with cell-surface sialylation of cultured murine tumor cell lines. *Science*, **212**, 1514.

YOGEESWARAN, G. & STEIN, B.S. (1980). Glycosphingolipids of metastatic variant RNA-virus-transformed nonproducer Balb 3T3 cell lines. *J. Natl Cancer Inst.*, **65**, 967.

YU, R.K. & LEDEEN, R.W. (1970). Gas-liquid chromatographic assay of lipid-bound sialic acid: measurement of gangliosides in brain of several species. *J. Lipid Res.*, **11**, 506.

YU, R.K. & LEDEEN, R.W. (1972). Gangliosides of human, bovine, and rabbit plasma. *J. Lipid Res.*, **13**, 680.